# Preoperative Phase Angle as a Marker of Nutritional and Functional Vulnerability in Patients Undergoing Colorectal Cancer Surgery: A Prospective Observational Cohort Study

**DOI:** 10.3390/nu18162619

**Published:** 2026-08-11

**Authors:** David Sánchez-Relinque, Alejandro García-García, M. Eugenia Valenzuela-Mateos, Jara Díaz-Jiménez, Álvaro Zambrano-Rico, María José M. Alférez, Eduardo Sánchez-Sánchez

**Affiliations:** 1 Unit of General and Digestive Surgery, Punta de Europa University Hospital, 11207 Algeciras, Spain; david.sanchez.relinque.sspa@juntadeandalucia.es (D.S.-R.); mariae.valenzuela.sspa@juntadeandalucia.es (M.E.V.-M.); 2 Biomedical Research and Innovation Institute of Cádiz (INiBICA), Puerta del Mar University Hospital, 11009 Cádiz, Spain; alejandro.garcia@inibica.es; 3 Department of Didactics of Physical, Plastic and Musical Education, University of Cádiz, 11519 Puerto Real, Spain; jara.diaz@uca.es; 4 Department of Nursing and Physiotherapy, University of Cádiz, 11009 Cádiz, Spain; alvaro.zambrano@uca.es; 5 Department of Physiology, Institute of Nutrition and Food Technology José Mataix, University of Granada, 18071 Granada, Spain; malferez@ugr.es

**Keywords:** colorectal neoplasms, phase angle, malnutrition, physical performance, postoperative complications, prehabilitation

## Abstract

**Background/Objectives**: This study evaluated whether phase angle (PhA) was associated more strongly with nutritional and functional vulnerability than with postoperative morbidity. **Methods**: This cohort included 207 adults undergoing colorectal cancer surgery. Primary outcomes were malnutrition defined according to the Global Leadership Initiative on Malnutrition (GLIM) criteria, low handgrip strength and impaired Short Physical Performance Battery (SPPB) scores; postoperative complications, readmissions and length of stay were exploratory outcomes. PhA was measured by 50 kHz bioelectrical impedance vector analysis. Analyses estimated areas under the curve (AUCs) with 95% confidence intervals and Youden cut-offs; models adjusted for age, sex and body mass index. **Results**: PhA was lower in participants with nutritional vulnerability than in those without it (4.57 ± 0.62° vs. 5.20 ± 0.69°; *p* < 0.001). Discrimination was excellent for handgrip strength <10th percentile (AUC 0.803) and acceptable for SPPB < 10 (AUC 0.785), SPPB ≤ 6 (AUC 0.761) and GLIM-defined malnutrition (AUC 0.728) but showed no discrimination for any postoperative complication (AUC 0.507). After adjustment, PhA ≤ 4.7° remained associated with GLIM-defined malnutrition, handgrip strength < 10th percentile and SPPB < 10 but not with any postoperative complication. **Conclusions**: PhA was more strongly associated with nutritional and functional vulnerability than with postoperative morbidity. Thresholds require external validation; PhA may complement, not replace, GLIM, body composition, handgrip strength and SPPB.

## 1. Introduction

Colorectal cancer surgery remains associated with morbidity despite minimally invasive techniques, anaesthetic optimisation and Enhanced Recovery After Surgery (ERAS) programmes [1]. Outcomes also depend on preoperative physiological reserve. Malnutrition, loss of muscle mass, reduced muscle strength and impaired physical performance may compromise the capacity to withstand surgery-related metabolic and inflammatory stress [2,3,4]. The Global Leadership Initiative on Malnutrition (GLIM) provides a standard framework for diagnosing malnutrition.

Early nutritional assessment and support are central to perioperative care. GLIM combines phenotypic and aetiologic criteria and provides updated guidance on muscle mass and inflammation assessment [5]. ERAS and clinical nutrition guidelines recommend early identification and management of malnutrition within multimodal perioperative pathways [1,3,6]. This is particularly relevant in colorectal cancer because malnutrition may coexist with overweight, obesity, lean-tissue depletion or fluid shifts that are not captured by body mass index alone [5,6].

Muscle mass reflects structural reserve, handgrip strength reflects neuromuscular function, and the Short Physical Performance Battery (SPPB) captures lower-extremity physical performance [4,7,8]. These domains are complementary rather than interchangeable and should be interpreted alongside standardised outcomes in multimodal prehabilitation [9].

Bioelectrical impedance analysis (BIA) is rapid and non-invasive. Phase angle (PhA), derived from resistance and reactance, reflects cell membrane integrity, fluid distribution and active body cell mass [10]. Its interpretation requires population-, device- and protocol-specific reference values [11]. Evidence from oncology scoping reviews, prospective studies, systematic reviews and meta-analyses links lower PhA to lean mass depletion, malnutrition, reduced handgrip strength, impaired physical function and poorer prognosis [12,13,14,15,16].

Evidence for postoperative outcomes is less consistent. In colorectal cancer surgery, Liu et al. incorporated PhA into a multivariable nomogram for postoperative complications [17]. Other reviews in cancer and surgical oncology report heterogeneous associations across populations, measurement protocols and outcome definitions [18,19]. The present study retained the clinically common “any postoperative complication” composite as a secondary exploratory endpoint to examine whether PhA discriminates broad morbidity. Because the composite combines events with different mechanisms and severity, a weaker association was expected than for nutritional and functional outcomes.

Accordingly, the primary objective was to evaluate baseline PhA as a marker of nutritional and functional vulnerability in adults with colorectal cancer scheduled for elective surgery within a multimodal prehabilitation pathway. Secondary objectives were to assess discrimination for any postoperative complication, readmission-related outcomes and prolonged hospital stay, and to explore outcome-specific PhA thresholds and quartile-based vulnerability gradients. We hypothesised that baseline PhA would be more strongly associated with GLIM-defined malnutrition, low handgrip strength and impaired physical performance than with broad composite postoperative morbidity.

## 2. Materials and Methods

### 2.1. Study Design

This prospective observational cohort study was reported in accordance with the Strengthening the Reporting of Observational Studies in Epidemiology (STROBE) guidelines [20].

### 2.2. Setting, Study Population and Analytical Sample

The study was conducted in the elective colorectal surgery pathway of the Campo de Gibraltar Oeste Healthcare Management Area, (Algeciras, Spain). Data were collected prospectively between September 2023 and May 2025. Consecutive adult patients with colorectal cancer scheduled for elective colorectal surgery were identified in preoperative surgical consultations.

Eligible participants were adults aged 18 years or older with a diagnosis of colorectal cancer, scheduled for elective colorectal surgery, and able to undergo baseline preoperative assessment of nutritional status, body composition and physical function before surgery.

Patients were excluded if they underwent emergency colorectal surgery, were unable to receive oral intake because of a clinical contraindication, or had digestive intolerance or an allergy to any component of the nutritional products used in the perioperative pathway.

The study size was fixed by the number of eligible consecutive patients recruited during the predefined September 2023–May 2025 data-collection period in the institutional pathway. No formal a priori sample-size calculation was performed because the cohort was established for real-world prospective recruitment rather than to confirm a minimum effect for each outcome. Precision was therefore assessed from event counts and 95% confidence intervals, and analyses with sparse events were treated as exploratory.

For the analytical cohort, patients were additionally excluded if baseline PhA data were invalid or unavailable or if 30-day postoperative follow-up was incomplete. The final analytical cohort therefore comprised patients with valid baseline PhA data, complete nutritional and functional assessments and sufficient postoperative follow-up.

### 2.3. Variables and Measurement Instruments

Baseline PhA was the main exposure. The primary analytical domain comprised GLIM-defined malnutrition, handgrip strength below the 10th percentile, handgrip strength below the 50th percentile, SPPB < 10 and SPPB ≤ 6. The secondary exploratory clinical domain comprised any postoperative complication, non-diarrhoeal complications, hospital readmission, readmission-related complications and hospital stay >5 days within 30 days. For descriptive comparisons, “baseline nutritional vulnerability” denotes the combined classification of malnutrition or nutritional risk; it is distinct from the specific outcome “GLIM-defined malnutrition”.

Potential confounding variables considered clinically relevant were age, sex, body mass index, tumour location, surgical approach, baseline nutritional status, body composition parameters and functional status. These variables were selected because of their potential association with both phase angle and postoperative or functional outcomes.

Body composition was assessed preoperatively using single-frequency bioelectrical impedance vector analysis at 50 kHz with a BIA 101 device (Akern, Florence, Italy) and a standardised tetrapolar technique. Measurements were obtained with the patient supine after 5 min of rest, with electrodes on the right hand and foot. Resistance and reactance were recorded and used to calculate PhA. Fat-free mass, fat mass, body cell mass, total body water, extracellular water, hydration percentage, appendicular muscle mass and skeletal muscle mass index were also obtained. The records available for this analysis did not document fasting, recent exercise or fluid-intake restrictions; hydration was therefore not standardised beyond the resting measurement protocol. Extracellular water and hydration percentage characterised fluid distribution; extracellular water was used only in the exploratory hydration-sensitivity models described below.

Preoperative nutritional status was assessed according to the GLIM consensus [5]. Malnutrition required at least one phenotypic criterion (weight loss, low body mass index or reduced muscle mass) and one aetiologic criterion (reduced intake or assimilation, inflammation or disease burden), and severity was classified using the GLIM criteria.

Muscle strength was assessed using a Jamar^®^ handgrip dynamometer (New York, NY, USA). Three attempts were performed according to a standardised protocol, and the mean value was used for analysis. Age- and sex-adjusted reference percentiles for the Spanish population were used for interpretation, according to Sánchez-Torralvo et al. [21]. Low muscle strength was defined as handgrip strength below the 10th percentile. In addition, handgrip strength below the 50th percentile was explored as an indicator of reduced muscle performance.

Physical performance was assessed using the Short Physical Performance Battery. An SPPB score < 10 was considered low physical performance, whereas an SPPB score ≤ 6 was considered marked functional impairment or vulnerability compatible with functional frailty.

The main exploratory clinical outcome was any postoperative complication within 30 days. This inclusive composite captured at least one recorded event of any listed type or severity, including diarrhoea; it was retained to reflect routine surveillance rather than a homogeneous pathophysiological endpoint. Non-diarrhoeal complications were therefore analysed separately. Other exploratory outcomes were readmission, readmission-related complications and hospital stay >5 days. Recorded events included diarrhoea, ileus, surgical site infection, intra-abdominal collection, suture or anastomotic dehiscence, phlebitis, intestinal ischaemia, bacteraemia, pneumonia, urinary tract infection, evisceration and atelectasis.

### 2.4. Data Collection and Follow-Up

**Figure 1:**
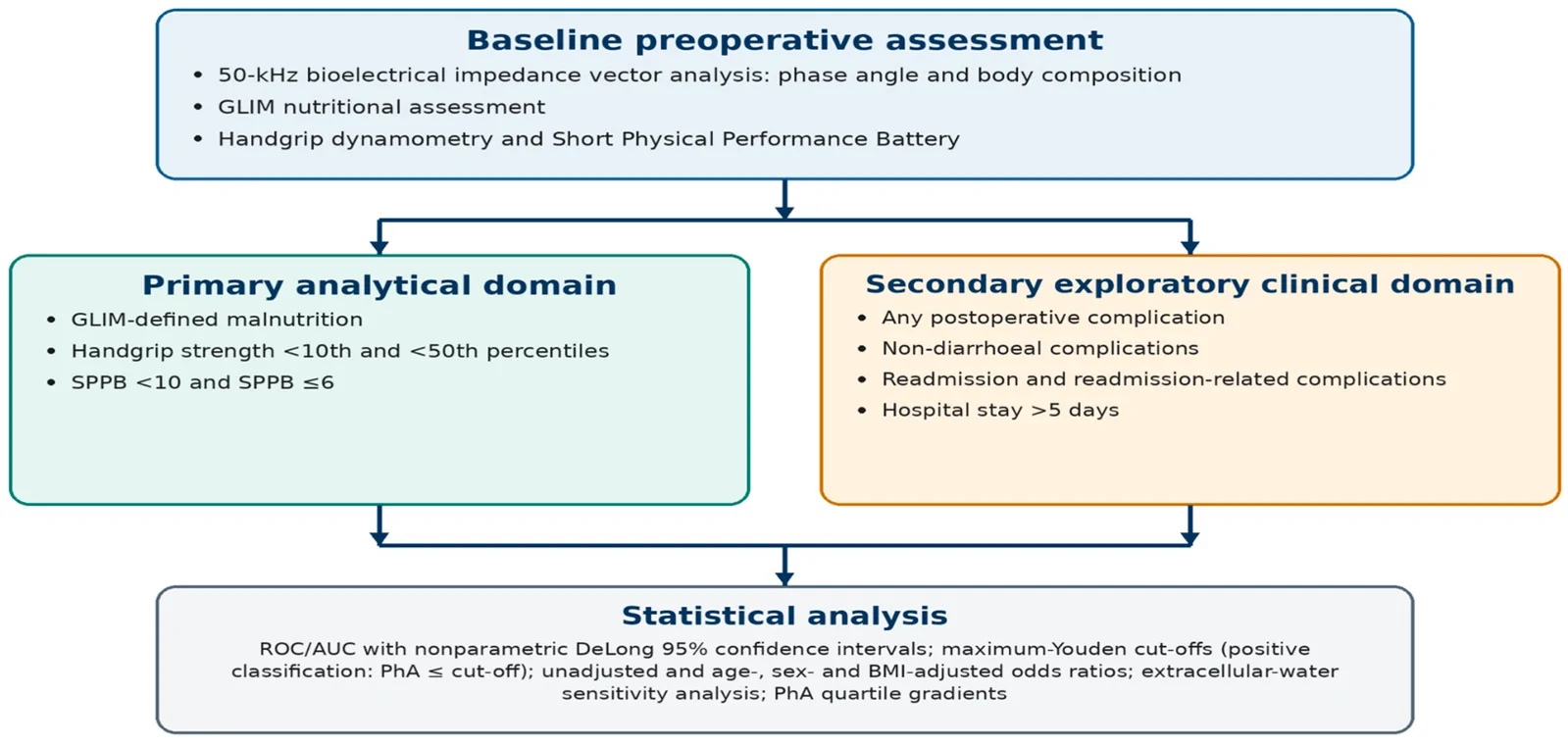
Schematic overview of the baseline assessments, outcome domains and analytical framework. PhA: phase angle; GLIM: Global Leadership Initiative on Malnutrition; SPPB: Short Physical Performance Battery; ROC: receiver operating characteristic; AUC: area under the curve; BMI: body mass index.

Baseline clinical, nutritional, functional and body composition variables were collected at the initial preoperative visit. Postoperative outcomes were recorded prospectively during hospital admission and up to 30 days after surgery. Three main time points for clinical assessment were considered: 24 h after surgery, on postoperative day 5 and at 30-day follow-up. Hospital readmissions, readmission-related complications and length of hospital stay were also recorded during the 30-day follow-up period. A schematic overview of the baseline assessments, outcome domains and analytical framework is provided in Figure 1.

### 2.5. Bias

Several measures were taken to reduce bias. Consecutive recruitment was used to limit clinician-related selection bias, and baseline assessments followed standardised procedures before surgery. Postoperative events and readmissions were collected prospectively using predefined clinical criteria. Formal blinding of outcome assessors to baseline PhA was not documented; blinded ascertainment therefore cannot be claimed, and detection bias remains possible.

Because this was an observational study, residual confounding cannot be excluded. Age, sex, body mass index, tumour location, surgical approach and baseline nutritional or functional status were considered potential sources of confounding because they may influence both phase angle and clinical outcomes. The interpretation of associations was therefore based not only on statistical significance but also on effect size, clinical coherence and biological plausibility.

### 2.6. Statistical Analysis

Continuous variables were summarised as means and standard deviations or medians and interquartile ranges, according to their distributions. Categorical variables were summarised as frequencies and percentages. The normality of continuous variables was assessed using the Shapiro–Wilk test.

Descriptive comparisons were performed using the chi-square test or Fisher’s exact test for categorical variables, and Student’s *t*-test or the Mann–Whitney U test for continuous variables, as appropriate.

The discriminative performance of baseline PhA was evaluated with receiver operating characteristic (ROC) curves. Areas under the curve (AUCs) and 95% confidence intervals (CIs) were estimated for each outcome using the nonparametric DeLong method. The threshold that maximised the Youden index (sensitivity + specificity − 1) was reported with its sensitivity, specificity and Youden J value. Because a lower PhA indicated greater vulnerability, positive low-PhA classifications were defined as PhA ≤ the selected cut-off. AUCs were interpreted as no discrimination (approximately 0.50), poor (0.60–0.69), acceptable (0.70–0.79) or excellent (0.80–0.89). Outcomes were GLIM-defined malnutrition, handgrip strength < 10th and <50th percentiles, SPPB < 10, SPPB ≤ 6, any postoperative complication, non-diarrhoeal complications, readmission, readmission-related complications and hospital stay >5 days.

Because 4.7° was the most frequently selected cut-off, it was applied as PhA ≤ 4.7° versus PhA > 4.7° to compare outcome frequencies and estimate unadjusted odds ratios (ORs) with 95% CIs. Exploratory multivariable logistic regression models then estimated adjusted odds ratios for PhA ≤ 4.7°, including age, sex and body mass index as covariates. This parsimonious adjustment set was selected before model fitting, no automated variable selection was used, and model dimensionality was limited because the least frequent primary outcome had 29 events. As a hydration-focused sensitivity analysis, extracellular water percentage was added to the adjustment set. Phase angle was also analysed by quartiles to explore gradients across increasing phase angle categories.

Potential confounders considered clinically relevant included age, sex, body mass index, tumour location, surgical approach, hydration, inflammation, comorbidities and medication use. The primary adjustment set was restricted to age, sex and body mass index to preserve complete-case sample size and avoid overfitting sparse endpoints. Extracellular water was added separately in the sensitivity analysis because it is closely related to fluid distribution and PhA. The models did not account simultaneously for all clinical factors; residual confounding remains, and the estimates are associative rather than causal.

The primary-domain ROC analyses and descriptive comparisons addressed the main analytical objective. Cut-off-derived odds ratios, quartile gradients and all postoperative outcome analyses were exploratory and hypothesis-generating. No subgroup or interaction analyses were prespecified, and no confirmatory multiplicity-adjusted testing framework was applied.

Missing data were handled with outcome-specific complete-case analyses. Readmission outcomes included 203 patients, and hospital stay >5 days included 199 patients. The reasons for these outcome-specific missing values were not documented in the analytical records available for this report; these missing values being completely random therefore cannot be assumed, and complete-case estimates may be biased. No imputation was performed.

Loss to follow-up was addressed by excluding patients without sufficient postoperative follow-up from the final analytical cohort. The number of participants excluded and the reasons for exclusion were reported in the participant flow description.

No post hoc power calculation was performed. Given the fixed sample and outcome-specific event counts, uncertainty was characterised with 95% confidence intervals. Comparisons with few events, particularly the 22 readmissions and 35 readmission-related complications, provided limited precision for multivariable effect estimation and were interpreted as hypothesis-generating. The adjusted models and extracellular water sensitivity analysis were exploratory. Statistical significance was set at *p* < 0.05.

### 2.7. Declaration of Generative AI and AI-Assisted Technologies in the Writing Process

During preparation of this work, the authors used OpenAI ChatGPT (GPT-5.5 version) for grammar checks, spelling correction, refinement of academic style and support in figure preparation. The tool was not used for study design, data collection, statistical analysis or primary interpretation of the results. The authors reviewed and edited all AI-assisted content and take full responsibility for the content of the manuscript.

## 3. Results

### 3.1. Baseline Characteristics and Preoperative Vulnerability Profile

During the study period, 220 patients entered the elective colorectal cancer pathway. One was excluded because baseline PhA was missing or invalid, and 12 because postoperative follow-up was insufficient for the main analytical cohort, leaving 207 participants. Participant flow, exclusions and outcome-specific data availability are shown in Figure S1.

Data for all primary-domain outcomes, any postoperative complication and non-diarrhoeal complications were available for all 207 participants. Readmission outcomes were available for 203 participants, and data on hospital stay >5 days were available for 199; complete-case analyses were performed for these secondary outcomes without imputation.

Baseline characteristics are shown in Table 1. Of 207 participants, 126 had no baseline nutritional vulnerability and 81 had baseline nutritional vulnerability (malnutrition or nutritional risk).

Compared with participants without baseline nutritional vulnerability, those with vulnerability had lower PhA (4.57 ± 0.62° vs. 5.20 ± 0.69°; *p* < 0.001), fat-free mass, body cell mass and skeletal muscle mass index, and higher extracellular water (Table 2).

They also had lower handgrip strength (27.21 ± 9.10 vs. 36.07 ± 11.50 kg; *p* < 0.001) and SPPB scores (6.36 ± 3.53 vs. 9.68 ± 2.26; *p* < 0.001). Moderate and severe GLIM-defined malnutrition was present in 87.7% and 1.6%, respectively.

### 3.2. Discriminative Performance of Baseline Phase Angle

**Figure 2:**
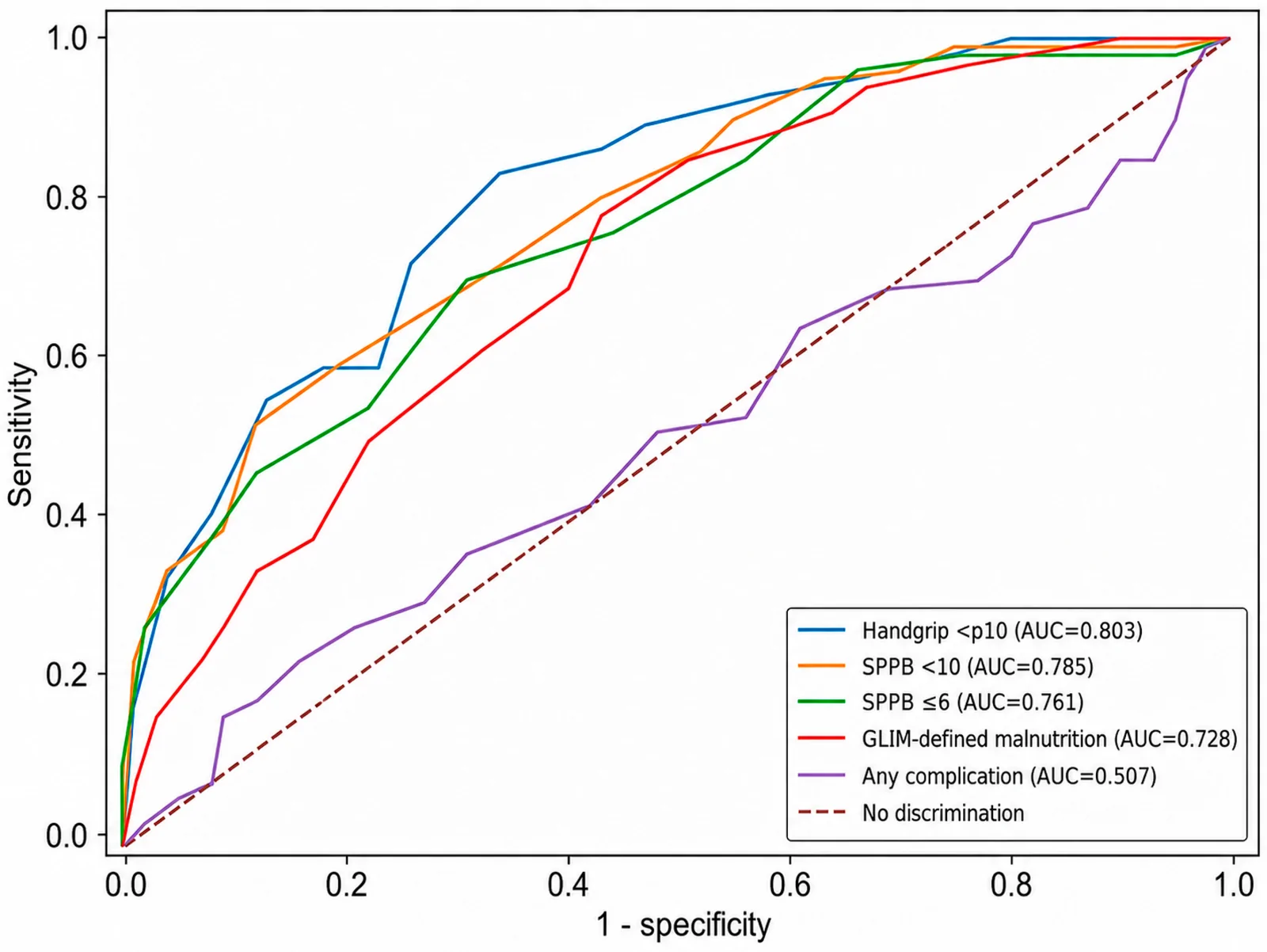
ROC curves of baseline phase angle for selected nutritional, functional and clinical outcomes. AUC estimates and nonparametric DeLong 95% confidence intervals are displayed in the legend. p10: 10th percentile; ROC: receiver operating characteristic; AUC: area under the curve; GLIM: Global Leadership Initiative on Malnutrition; SPPB: Short Physical Performance Battery.

ROC curves for selected outcomes are shown in Figure 2. The 95% confidence intervals for each AUC are displayed in the figure legend and reported for all outcomes in Table 3.

Using the prespecified terminology, discrimination was excellent for handgrip strength <10th percentile (AUC 0.803) and acceptable for SPPB < 10 (0.785), SPPB ≤ 6 (0.761) and GLIM-defined malnutrition (0.728). Compared with any postoperative complication (AUC 0.507), the corresponding AUC differences were 0.296, 0.278, 0.254 and 0.221.

PhA showed no useful discrimination for any postoperative complication (AUC 0.507) or non-diarrhoeal complications (AUC 0.499). Discrimination for readmission, readmission-related complications and hospital stay >5 days was poor (AUCs 0.613–0.631) and imprecise (Table 3).

### 3.3. Clinical Interpretation of the Phase Angle Threshold

According to the maximum Youden index, 4.7° was the most frequently selected cohort-derived threshold for functional outcomes, whereas 5.0° was selected for GLIM-defined malnutrition. These values are candidate screening thresholds requiring external validation and should not be interpreted as prespecified or universal clinical action thresholds.

All five nutritional and functional outcomes were more frequent among participants with PhA ≤ 4.7°, with unadjusted ORs ranging from 3.32 for GLIM-defined malnutrition to 8.98 for handgrip strength <10th percentile (Table 4).

In contrast, PhA ≤ 4.7° was not associated with any postoperative complication or non-diarrhoeal complications. Readmission and readmission-related complications showed elevated unadjusted estimates, whereas hospital stay >5 days did not; these complete-case findings were exploratory and imprecise.

After adjustment for age, sex and body mass index, associations remained for handgrip strength <10th percentile (adjusted OR 5.67, 95% CI 1.82–17.66), handgrip strength < 50th percentile (3.35, 1.70–6.58) and SPPB < 10 (3.01, 1.49–6.05). The estimates for GLIM-defined malnutrition and SPPB ≤ 6 were weaker or imprecise.

No adjusted association was observed for any postoperative complication, non-diarrhoeal complications, readmission or hospital stay >5 days. The adjusted estimate for readmission-related complications remained elevated but was based on only 35 events. Adding extracellular water attenuated the dichotomised PhA estimates.

In exploratory base-model comparisons, adding continuous PhA to models containing age, sex and body mass index increased the AUC by 0.028–0.058 for the primary nutritional and functional outcomes, whereas the AUC for any postoperative complication was unchanged (0.611 versus 0.611). These internal comparisons were not optimism-corrected.

### 3.4. Vulnerability Gradient According to Phase Angle Quartiles

Quartile analyses were exploratory and assessed whether nutritional, functional or clinical outcomes showed a monotonic pattern across increasing PhA categories.

Median PhA increased from 4.2° [3.9–4.3] in Q1 to 5.7° [5.6–6.3] in Q4 (Table 5).

From Q1 to Q4, GLIM-defined malnutrition decreased from 57.7% to 9.8%, and handgrip strength < 10th percentile decreased from 32.7% to 2.0%.

**Figure 3:**
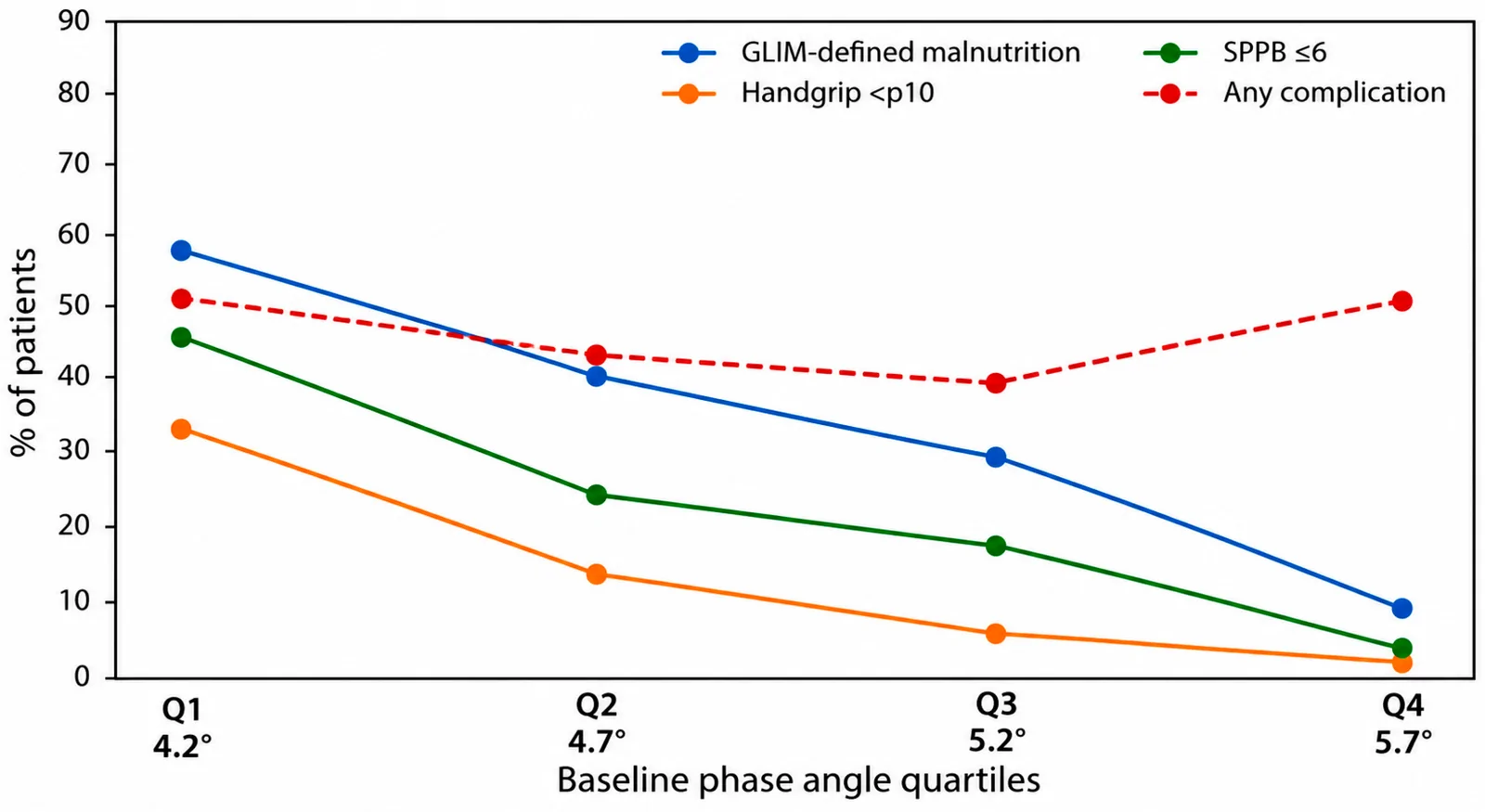
Gradients of nutritional and functional vulnerability across baseline phase angle quartiles. GLIM: Global Leadership Initiative on Malnutrition; SPPB: Short Physical Performance Battery.

SPPB ≤ 6 decreased from 46.2% to 3.9%, and SPPB < 10 from 82.7% to 17.6%. The frequency of any postoperative complication did not show a monotonic gradient (51.9%, 44.2%, 40.4% and 51.0% across Q1–Q4; Figure 3).

The absence of a monotonic gradient for any postoperative complication is displayed in Table 5 and Figure 3.

## 4. Discussion

Baseline PhA identified nutritional and functional vulnerability but did not discriminate the heterogeneous composite of any postoperative complication. This is an outcome-specific limitation, not evidence that PhA is uniquely inferior to established surgical risk measures. Because the American Society of Anesthesiologists Physical Status Classification System (ASA-PS), the Physiological and Operative Severity Score for the enUmeration of Mortality and morbidity (POSSUM), and other established surgical risk scores were not compared head-to-head in this cohort, no claims about relative performance can be made.

This pattern is biologically plausible. Lower PhA reflects lower reactance relative to resistance and may indicate impaired cell membrane integrity, reduced body cell mass and extracellular fluid expansion [10,18]. Malnutrition, systemic inflammation and muscle catabolism can produce these changes and may reduce muscle contractile function, immune competence and tissue repair [22,23,24,25]. In contrast, postoperative events also depend on operative complexity, technical factors, infectious exposures and care processes, which weakens the relationship with a single baseline bioelectrical marker.

Our findings align with reviews and prospective oncology studies linking lower PhA with malnutrition, lower handgrip strength, slower gait, sarcopenia and poorer prognosis [12,13,14,15,16,18,24]. They also support treating nutritional, functional and clinical endpoints as distinct rather than interchangeable.

Previous surgical studies are not directly comparable. Liu et al. incorporated PhA into a multivariable nomogram [17], while Gulin et al. studied a smaller heterogeneous gastrointestinal cancer cohort and reported a 5.5° cut-off for graded complications [26]. In our cohort, the 46.9% rate reflects an inclusive composite that included diarrhoea and events of varying severity; the non-diarrhoeal rate was 22.7%. Direct comparison with cohorts reporting only graded or severe morbidity is therefore inappropriate.

The 5.0° and 4.7° cut-offs were selected separately by the Youden index for GLIM-defined malnutrition and functional vulnerability. Published oncology cut-offs vary by population, device, frequency and endpoint; for example, Gulin et al. reported 5.5° for postoperative complications [26], while reviews document broad variation and no universal standard [15,16,27]. Our values should therefore be described as cohort-derived candidate screening thresholds, not treatment thresholds.

PhA should complement rather than replace standard nutritional and functional assessments. In exploratory comparisons, adding continuous PhA to models containing age, sex and body mass index increased the AUCs for the primary nutritional and functional outcomes but not for any postoperative complication. This does not establish incremental value beyond GLIM, handgrip strength, SPPB, ASA-PS, POSSUM or other established assessments because head-to-head combined models, calibration, reclassification and effects on clinical decision-making were not evaluated. PhA may serve as a rapid trigger for completing recommended assessments when they are not yet available. Future studies should compare PhA alone, established measures and validated combined approaches.

Hydration, inflammation, comorbidities and medication use may influence both PhA and postoperative outcomes. Extracellular water and hydration percentage were measured, but hydration was not standardised beyond supine rest. Adding extracellular water to the sensitivity models adjusted for age, sex and body mass index attenuated the dichotomised PhA estimates, indicating material hydration-related confounding or collinearity. Because extracellular water is mechanistically coupled to the bioelectrical signal, this sensitivity analysis does not isolate a direct PhA effect. Diuretics, corticosteroids, intravenous fluids and inflammatory burden could also alter fluid distribution or cellular reactance and were not modelled.

Within ERAS pathways, low PhA should prompt or reinforce the nutritional and functional assessments that are already recommended, not replace them or define a new independent standard of care [1,3,28]. No trial cited here selected or treated patients solely according to a low PhA threshold; therefore, improved outcomes from a PhA-guided intervention cannot be claimed.

Multimodal prehabilitation can improve functional recovery in selected colorectal surgery populations, but effects vary by modality, adherence and outcome [2,29,30,31]. We measured PhA only at baseline. Serial perioperative changes were not analysed, so this study cannot determine whether changes in PhA track recovery or mediate intervention effects. Prospective studies with repeated measurements are needed before PhA can be used to monitor response.

The strengths of this study include its prospective design, consecutive recruitment, a cohort of 207 participants, and the integration of body composition, GLIM criteria, muscle strength and physical performance. In addition, the analysis combined ROC curves, cut-offs and quartiles, allowing a more comprehensive clinical interpretation than that based on a single AUC estimate. The outcome-specific interpretation also avoids treating PhA as a universal predictor and instead places its potential role within preoperative nutritional and functional assessment.

Limitations include the single-centre design, a fixed sample recruited over a predefined period and the absence of an a priori sample-size calculation. Secondary clinical analyses had few events and wide confidence intervals and were exploratory. Complete-case analyses excluded four participants with missing readmission data and eight with missing hospital-stay data; the reasons for missing data were not documented, so selection bias cannot be excluded. Although exploratory models adjusted for age, sex and body mass index, residual confounding remains because tumour and operative factors, inflammation, comorbidities and medication effects were not modelled. Extracellular water sensitivity models were potentially affected by collinearity with PhA. Incremental value beyond established nutritional, functional or surgical-risk measures was not tested. Blinding of outcome assessors was not documented, and hydration was not fully standardised. The inclusive any-complication composite mixed events of different mechanisms and severity.

The findings apply primarily to adults undergoing elective colorectal cancer surgery within a structured perioperative pathway. Excluding emergency cases and patients unable to use the oral route may have underrepresented the most vulnerable patients, thereby limiting generalisability. Results should not be extrapolated to those groups or to settings without standardised assessments. Variations related to the device, protocol, population and hydration status also necessitate external validation of any cut-off.

## 5. Conclusions

Among patients undergoing elective colorectal cancer surgery, lower preoperative PhA was associated with GLIM-defined malnutrition, reduced handgrip strength and impaired physical performance in models adjusted for age, sex and BMI, although these associations were attenuated after additional adjustment for extracellular water. By contrast, PhA provided little discrimination for the broad composite of any postoperative complication. The cohort-derived values of 5.0° for GLIM-defined malnutrition and 4.7° for functional vulnerability should be regarded as outcome-specific candidate screening thresholds rather than universal clinical thresholds. PhA may therefore serve as a complementary marker that prompts comprehensive nutritional and functional assessment alongside GLIM, body composition, handgrip strength and SPPB. External validation and formal evaluation of its incremental clinical value are required before routine implementation.

## Figures and Tables

**Table 1:** Baseline demographic, clinical, nutritional, body composition and functional characteristics of the final analytical cohort.

Variable	Final Analytical Cohort (*n* = 207)
Age, years	67.98 ± 11.58
Sex, *n* (%) Female Male	83 (40.1) 124 (59.9)
Tumour location, *n* (%) Colon cancer Rectal cancer Other/unspecified colorectal location	168 (81.2) 34 (16.4) 5 (2.4)
BMI, kg/m^2^	26.94 ± 4.82
Phase angle, °	4.95 ± 0.73
FFM, kg	31.46 ± 4.83
BCM, kg	15.01 ± 3.23
SMI, kg/m^2^	8.65 ± 1.73
ECW, %	51.19 ± 4.83
Handgrip strength, kg	32.60 ± 11.45
Total SPPB	8.38 ± 3.26
Moderate or severe GLIM-defined malnutrition	73 (35.3)

Values are expressed as mean ± standard deviation or n (%). BCM: body cell mass; BMI: body mass index; ECW: extracellular water; FFM: fat-free mass; GLIM: Global Leadership Initiative on Malnutrition; SMI: skeletal muscle mass index; SPPB: Short Physical Performance Battery.

**Table 2:** Baseline body composition and functional profile according to baseline nutritional vulnerability.

Variable	No Baseline Nutritional Vulnerability (*n* = 126)	Baseline Nutritional Vulnerability (*n* = 81)	*p*-Value
Phase angle, °	5.20 ± 0.69	4.57 ± 0.62	<0.001
FFM, kg	32.58 ± 4.85	29.72 ± 4.27	<0.001
BCM, kg	15.98 ± 3.35	13.48 ± 2.33	<0.001
SMI, kg/m^2^	8.85 ± 1.64	8.34 ± 1.84	0.044
ECW, %	49.70 ± 4.65	53.51 ± 4.15	<0.001
Handgrip strength, kg	36.07 ± 11.50	27.21 ± 9.10	<0.001
Total SPPB	9.68 ± 2.26	6.36 ± 3.53	<0.001
Moderate or severe GLIM-defined malnutrition	2 (1.6)	71 (87.7)	< 0.001

Values are expressed as mean ± standard deviation or n (%). *p* values compare participants with and without baseline nutritional vulnerability (malnutrition or nutritional risk). BCM: body cell mass; ECW: extracellular water; FFM: fat-free mass; GLIM: Global Leadership Initiative on Malnutrition; SMI: skeletal muscle mass index; SPPB: Short Physical Performance Battery.

**Table 3:** Discriminative performance of baseline phase angle for nutritional, functional and clinical outcomes.

Outcome	*n*/Events	AUC (95% CI)	Low-PhA Cut-Off, °	Sensitivity, %	Specificity, %	Youden J
GLIM-defined malnutrition	207/73	0.728 (0.660–0.797)	≤5.0	78.1	57.5	0.355
Handgrip strength < 10th percentile	207/29	0.803 (0.720–0.887)	≤4.7	82.8	65.2	0.479
Handgrip strength < 50th percentile	207/96	0.715 (0.644–0.786)	≤4.7	61.5	75.7	0.371
SPPB < 10	207/108	0.785 (0.725–0.846)	≤4.7	60.2	78.8	0.390
SPPB ≤ 6	207/48	0.761 (0.683–0.839)	≤4.7	70.8	67.3	0.381
Any postoperative complication	207/97	0.507 (0.426–0.587)	≤4.1	16.5	90.9	0.074
Any non-diarrhoeal complication	207/47	0.499 (0.395–0.603)	≤4.4	34.0	78.8	0.128
Readmission	203/22	0.613 (0.478–0.748)	≤4.7	63.6	60.8	0.244
Readmission-related complications	203/35	0.631 (0.525–0.738)	≤4.7	65.7	63.1	0.288
Hospital stay >5 days	199/67	0.613 (0.527–0.699)	≤4.5	41.8	78.8	0.206

AUC: area under the ROC curve; CI: confidence interval; GLIM: Global Leadership Initiative on Malnutrition; SPPB: Short Physical Performance Battery. AUC confidence intervals were calculated using the nonparametric DeLong method. Low-PhA cut-offs were selected according to the maximum Youden index (J = sensitivity + specificity − 1), with a positive classification defined as PhA ≤ the reported cut-off.

**Table 4:** Unadjusted and adjusted associations of baseline phase angle ≤4.7° with nutritional and functional vulnerability and clinical outcomes.

Outcome	PhA ≤ 4.7°	PhA > 4.7°	Unadjusted OR (95% CI)	Adjusted OR (95% CI)
Nutritional outcome				
GLIM-defined malnutrition	44/86 (51.2)	29/121 (24.0)	3.32 (1.83–6.02)	1.99 (1.00–3.97)
Functional outcomes				
Handgrip strength < 10th percentile	24/86 (27.9)	5/121 (4.1)	8.98 (3.27–24.70)	5.67 (1.82–17.66)
Handgrip strength < 50th percentile	59/86 (68.6)	37/121 (30.6)	4.96 (2.73–9.02)	3.35 (1.70–6.58)
SPPB < 10	65/86 (75.6)	43/121 (35.5)	5.61 (3.03–10.41)	3.01 (1.49–6.05)
SPPB ≤ 6	34/86 (39.5)	14/121 (11.6)	5.00 (2.47–10.11)	2.12 (0.95–4.77)
Exploratory clinical outcomes				
Any postoperative complication	41/86 (47.7)	56/121 (46.3)	1.06 (0.61–1.84)	0.99 (0.52–1.89)
Any non-diarrhoeal complication	22/86 (25.6)	25/121 (20.7)	1.32 (0.69–2.54)	1.44 (0.67–3.08)
Readmission	14/85 (16.5)	8/118 (6.8)	2.71 (1.08–6.79)	2.60 (0.92–7.38)
Readmission-related complications	23/85 (27.1)	12/118 (10.2)	3.28 (1.52–7.04)	4.23 (1.70–10.52)
Hospital stay >5 days	34/83 (41.0)	33/116 (28.4)	1.75 (0.96–3.16)	1.54 (0.77–3.08)

Denominators vary for readmission-related outcomes and hospital stay because outcome-specific complete-case analyses were performed. Values are expressed as *n* (%). PhA: phase angle; GLIM: Global Leadership Initiative on Malnutrition; CI: confidence interval; OR: odds ratio; SPPB: Short Physical Performance Battery. Adjusted ORs are from exploratory logistic regression models including age, sex and body mass index. Sparse clinical events limit precision.

**Table 5:** Clinical, nutritional and functional gradients according to baseline phase angle quartiles.

Variable	Q1 (*n* = 52)	Q2 (*n* = 52)	Q3 (*n* = 52)	Q4 (*n* = 51)
Phase angle, °	4.2 [3.9–4.3]	4.7 [4.6–4.8]	5.2 [5.1–5.2]	5.7 [5.6–6.3]
GLIM-defined malnutrition	30 (57.7)	22 (42.3)	16 (30.8)	5 (9.8)
Handgrip strength < 10th percentile	17 (32.7)	8 (15.4)	3 (5.8)	1 (2.0)
Handgrip strength < 50th percentile	37 (71.2)	28 (53.8)	17 (32.7)	14 (27.5)
SPPB < 10	43 (82.7)	31 (59.6)	25 (48.1)	9 (17.6)
SPPB ≤ 6	24 (46.2)	13 (25.0)	9 (17.3)	2 (3.9)
Any postoperative complication	27 (51.9)	23 (44.2)	21 (40.4)	26 (51.0)
Any non-diarrhoeal complication	16 (30.8)	9 (17.3)	9 (17.3)	13 (25.5)
Readmission-related complications	14 (27.5)	11 (21.2)	4 (7.8)	6 (12.2)
Hospital stay >5 days	25 (51.0)	17 (32.7)	13 (25.5)	12 (25.5)

Values are expressed as median [interquartile range] or *n* (%). GLIM: Global Leadership Initiative on Malnutrition; SPPB: Short Physical Performance Battery.

## Data Availability

The data supporting the findings of this study are not publicly available due to privacy and ethical restrictions related to the use of individual-level clinical data. De-identified data may be made available from the corresponding author upon reasonable request, subject to approval by the relevant ethics committee and institutional data-sharing policies. No directly identifiable participant data will be shared.

## Supplementary Materials

The following supporting information can be downloaded at: https://www.mdpi.com/article/10.3390/nu18162619/s1, Figure S1: Participant flow and outcome-specific data availability.

## Abbreviations

The following abbreviations are used in this manuscript:

AUC	Area under the receiver operating characteristic curve
ASA-PS	*American Society of Anesthesiologists Physical Status Classification System*
BIA	Bioelectrical impedance analysis
BMI	Body mass index
CI	Confidence interval
ERAS	Enhanced recovery after surgery
INiBICA	Biomedical Research and Innovation Institute of Cádiz
FFM	Fat-free mass
BCM	Body cell mass
ECW	Extracellular water
GLIM	Global Leadership Initiative on Malnutrition
OR	Odds ratio
PhA	Phase angle
POSSUM	Physiological and Operative Severity Score for the enUmeration of Mortality and morbidity
ROC	Receiver operating characteristic
STROBE	Strengthening the Reporting of Observational Studies in Epidemiology
SMI	Skeletal muscle mass index
SPPB	Short Physical Performance Battery
