# Supplementary material for: Preoperative Phase Angle as a Marker of Nutritional and Functional Vulnerability in Patients Undergoing Colorectal Cancer Surgery: A Prospective Observational Cohort Study
Source: Nutrients. 2026 Aug 11;18(16):2619. doi: 10.3390/nu18162619 (PMC13516681; doi:10.3390/nu18162619)

Figure S1. STROBE 2025. Flow Diagram.

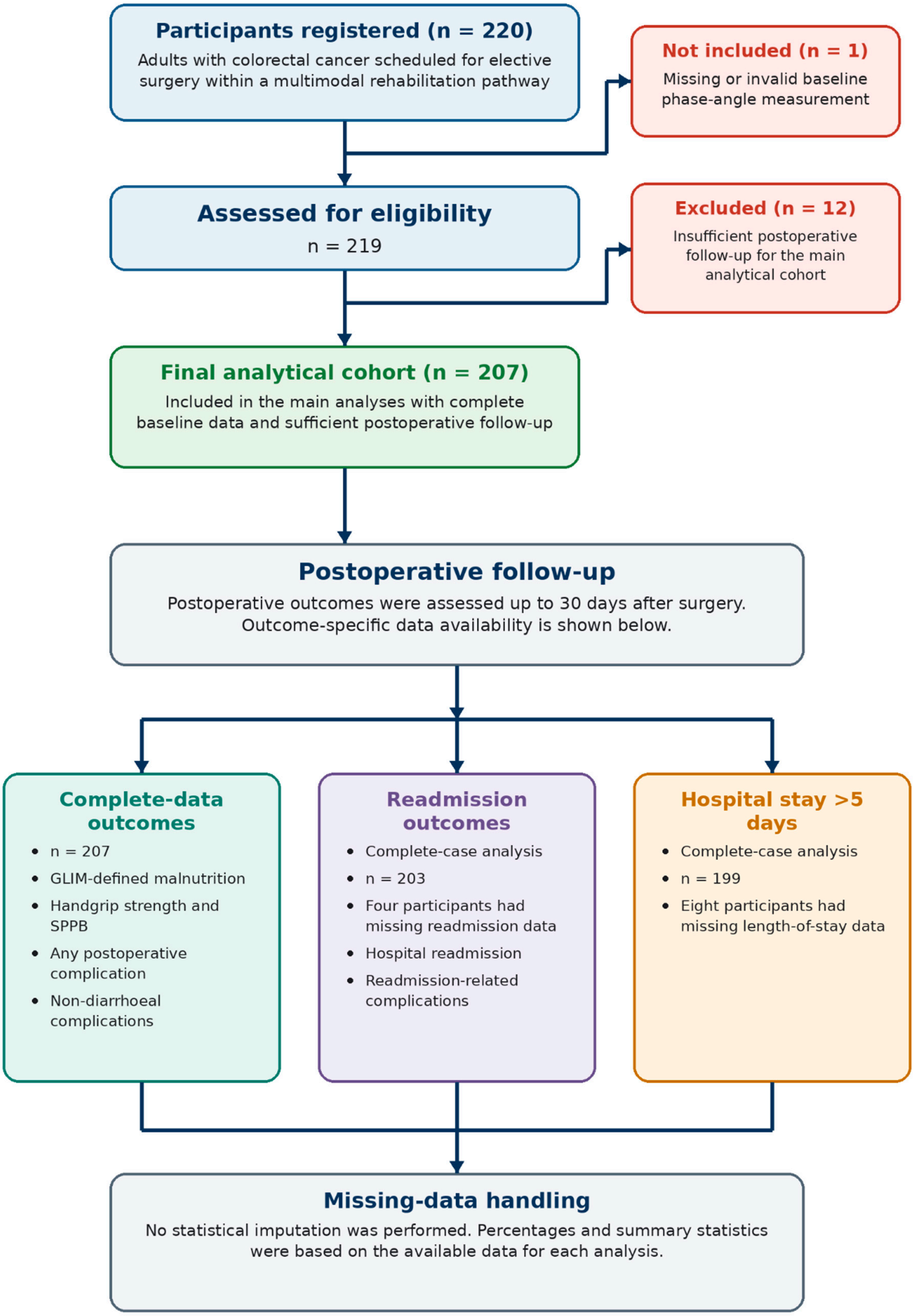

Supplement: Supplementary file 1 [file nutrients-18-02619-s001.zip › nutrients-4460585-supplementary.pdf]
